# WISP1 mediates hepatic warm ischemia reperfusion injury via TLR4 signaling in mice

**DOI:** 10.1038/srep20141

**Published:** 2016-01-29

**Authors:** Yao Tong, Xi-Bing Ding, Zhi-Xia Chen, Shu-Qing Jin, Xiang Zhao, Xin Wang, Shu-Ya Mei, Xi Jiang, Lingyu Wang, Quan Li

**Affiliations:** 1 Department of Anesthesiology, Shanghai East Hospital, School of Medicine, Tongji University, Shanghai 200120, China; 2 Department of Anesthesiology, First Clinical College of Nanjing Medical University, Nanjing 210029, Jiangsu, China; 3 Department of Anesthesiology, School of Medicine, Nanchang University, Nanchang 330031, Jiangxi, China

## Abstract

Wnt-induced secreted protein-1 (WISP1) is an extracellular matrix protein that has been reported in cancer researches. Our previous studies on WISP1 implied it could be a harmful mediator in septic mice. However, its role in liver ischemia reperfusion (I/R) injury is unknown. This study investigated the effects of WISP1 on liver I/R damage. Male C57BL/6 wild-type mice were used to undergo 60 min segmental (70%) ischemia. WISP1 expression was measured after indicated time points of reperfusion. Anti-WISP1 antibody was injected intraperitoneally to mice. Toll-like receptor 4 (TLR4) knockout mice and TIR-domain-containing adaptor inducing interferon-β (TRIF) knockout mice were adopted in this study. WISP1 was significantly enhanced after 6 h of reperfusion when compared with sham treated mice and significantly decreased either by TLR4 knockout mice or TRIF knockout mice. Anti-WISP1 antibody significantly decreased serum alanine aminotransferase (ALT), aspartate aminotransferase (AST), pathological changes and pro-inflammatory cytokine levels in the mice following I/R. Furthermore, significantly increased serum transaminase levels were found in C57 wild-type mice treated with recombinant WISP1 protein, but not found in TLR4 knockout or TRIF knockout mice subjected to liver I/R. Taken together, WISP1 might contribute to hepatic ischemia reperfusion injury in mice and possibly depends on TLR4/TRIF signaling.

Hepatic ischemia reperfusion (I/R) injury is unavoidable during shock, trauma, elective liver resections or liver transplantation, and adversely affects patient^1^,^2^. It concludes the two interrelated phases of local ischemia insult and inflammation-mediated reperfusion injury^3^. Hepatic I/R injury involves warm and cold ischemia. Warm ischemia occurs when the organism undergo trauma, shock and elective liver resections that liver blood supply is temporarily disrupted^4^. Cold ischemia occurs during liver preservation following by transplantation^5^. Various mediators, such as tumor necrosis factor receptor-associated factor 1 (TRAF1), tumor necrosis factor (TNF)-α, high-mobility group box 1 (HMGB1), and chemokines, contribute to hepatic I/R injury^6^,^7^,^8^,^9^. In order to improve the life quality of patients who suffered from liver I/R damage, it is essential to explore other potential harmful factors and find therapeutic methods to prevent liver dysfunction.

Wnt-induced secreted protein-1 (WISP1) is a secreted extracellular matrix protein, also known as a member of CCN family (CCN4), which can initiate the activation of signal transduction pathways through cell surface receptors for regulating diverse cellular functions^10^. WISP1 mRNA was found in several organs, such as lung, heart, kidney, liver and placenta. Former studies about WISP1 mainly devoted to tumor, multi-organ fibrosis and airway remodeling^11^,^12^,^13^,^14^. In addition, we and others have uncovered WISP1 can also contribute to ventilator induced acute lung injury or sepsis^15^,^16^. WISP1 could be an endogenous harmful factor participates in the pathogenesis of some acute diseases. Various harmful factors have been demonstrated in liver I/R injury. However, whether WISP1 acts as a harmful mediator involves in liver I/R injury is still unknown.

TLR4 is one of the pathogen recognition receptors (PRRS), which locates in the interface of microbial and sterile inflammation by targeting either bacterial endotoxin or some kinds of endogenous ligands, involving fibronectin, heparan sulphate, Heat shock proteins and HMGB1^17^,^18^,^19^,^20^,^21^. Liver subjected to ischemia reperfusion occurs inflammatory injury response possibly depends on TLR4^22^,^23^. Two key adaptor molecules, myeloid differentiation factor 88 (MyD88) and TRIF, which contain Toll/IL-1 Receptor (TIR) domains that can directly interact with TLRs, were located in the downstream of TLR4. Importantly, Zhai *et al.* have uncovered that TLR4-mediated liver I/R injury appears to be independent of MyD88 signaling, but dependent of TRIF signaling^24^.

Endogenous TLR4 ligands have pivotal roles in liver I/R injury^25^. The aim of this study was to determine whether WISP1 contributes to hepatic I/R injury and depends on TLR4/TRIF signal pathway in a mouse model of I/R injury.

## Materials and Methods

### Reagents

Anti-WISP1 monoclonal antibody, isotype control IgG and recombinant WISP1 protein were purchased from R&D Systems (Minneapolis, MN, USA).

### Animals

Male wild-type mice (C57BL/6; 8-12 weeks old) were purchased from Shanghai Laboratory Animal Co Ltd (SLAC, Shanghai, China). TLR4 knockout (TLR4 KO), TRIF KO and MyD88 KO mice were kindly provided by Dr. Timothy R. Billiar (University of Pittsburgh, USA). All mice were fed in a laminar-flow, specific pathogen-free atmosphere at the Shanghai Tongji University. Animal protocols were approved by the Ethics Committee of the University of Tongji and the experiments were performed in accordance with the National Institutes of Health Guidelines for the Use of Laboratory Animals.

### Experimental design

Mice partial (70%) warm ischemia reperfusion (I/R) injury model was performed as previously described^23^. Mice received anti-WISP1 monoclonal antibody (1 μg/g, 2 μg/g or 6 μg/g) or isotype control IgG via intraperitoneal (i.p.) injection 1 h before ischemia and again at the time of reperfusion, or recombinant WISP1 protein (20 μg per mouse) or sterile PBS i.p. immediately after reperfusion. Sham mice were sufficiently anesthetized, and then a midline abdominal incision was made, but only exposure of the portal triad without liver ischemia. Mice were sacrificed at the predetermined time points (0 h, 3 h, 6 h, 12 h and 24 h) after reperfusion for serum and liver samples.

### Liver damage assessment

To detect liver function and cellular injury following liver I/R injury, sALT and sAST levels were measured using the automated clinical analyzer (OLYMPUS AU1000; Olympus, Tokyo, Japan).

### Histopathology

Mice liver samples were collected, stored in 4% paraformaldehyde and embedded in paraffin. Paraffin Sections (5 um thick) were stained with hematoxylin-eosin (H&E) and observed by light microscopy; the assessment of numerical graduation of cell necrosis was as follows: 0 = nonnecrotic cells, 1 = single-cell necrosis, 2 = <30% necrosis, 3 = <60% necrosis, and 4 = >60% necrosis^26^. The pathology slides were reviewed by three observers blinded to the treatment group.

### Immunohistochemical staining

Liver sections to measure WISP1 were performed in 4% paraformaldehyde overnight. Sections were then embedded and cut into 5-um, which were placed onto slides. These slides were heated at 67 °C for 30 min and dewaxed in dimethylbenzene. Slides were then dehydrated in a concentration gradient of alcohol and pretreated with microwave heat-induced eritope retrieval. After that, slides were incubated with the primary antibody (anti-WISP1) at 1:100 dilution for 24 h at 4 °C and then the secondary antibody at 1:50 dilution was applied for 1 h at 28 °C. Slides were stained by diaminobenzidine and then visualized using a digital camera (Olympus) combined with light microscope at ×200 magnification.

### Isolation of Kupffer Cells

Mice kupffer cells were isolated according to the previous studies have reported^27^,^28^. Mice were anesthetized by ketamine (100 mg/kg) and xylazine (10 mg/kg), then livers were perfused with 0.05% Collagenase *in vivo* and digested with 0.04% Collagenase at 37 °C for additional 20 minutes *in vitro* and passed through a 250 μm cell strainer. After separation by a 25% and 50% Percoll gradient centrifugation for one hour at 800 ×  g at 4 °C, non-parenchymal liver cells were collected from the interface and cultured in Dulbecco’s modified eagle medium +10% fetal bovine serum; the nonadherent fraction was washed and the adherent Kupffer cells were remained.

### RNA interference

Small interfering RNA (siRNA) targeting mouse *WISP1* was synthesized by GIMA. siRNA were transfected into kupffer cells using siPORT NeoFX transfection reagents (Invitrogen) according to the standard protocol. The knockdown efficacy was detected by RT-PCR or Western blot.

### SDS-PAGE and Western blot analysis

Proteins were extracted from the frozen liver tissues by grinding with protease inhibitors. The proteins were incubated in boiling water for 15 min before the experiment. The protein samples were separated by 10% sodium dodecyl sulfate polyacrylamide gel electrophoresis (SDS-PAGE) and transferred onto a Polyvinylidene Fluoride (PVDF) membrane. The membranes were incubated with 5% nonfat milk for 1 h, and then incubated with primary antibodies. Primary rabbit monoclonal antibody to WISP1 (R&D Systems, Minneapolis, MN) was used for Western blot analysis. Membranes were detected using the Odyssey Two-Color Infrared Laser Imaging System.

### SYBR green real-time RT-PCR

Extraction of total RNA in liver tissues from mice who subjected ischemic and non-ischemic was performed by using TRIzol Reagent (Takara, Shiga, Japan). Total RNA was reversed transcribed into cDNA via using the SYBR Premix Ex Taq kit (TaKaRa Biotechnology China). SYBR Green quantitative RT-PCR was prepared to measure the expression of the target genes. The PCR primers used for this study showed as follows: WISP1 (F: 5′-CAGCACCACTAGAGGAAACGA-3′; R: 5′- CTGGGCACATATCTTACAGCATT-3′), IL-6 (F: 5′-TAGTCCTTCCTACCCCAATTTCC-3′; R: 5′-TTGGTCCTTAGCCACTCCTTC-3′), TNF-α (F: 5′-TAGCAAACCACCAAGTG-3′; R: 5′-ACAAGGTACAACCCATCG),

β-actin (F: 5′- GGCTGTATTCCCCTCCATCG-3′; R: 5′- CCAGTTGGTAACAATGCCATGT-3′). IL-1β (F: 5′-AACCTCACCTACAGGGCGGACTTCA-3′; R: 5′-TGTAATGAAAGACGGCACACC-3′)

iNOS (F: 5′-CCCTTCCGAAGTTTCTGGCAGCAGCG-3′; R: 5′-GGCTGTCAGAGCCTCGTGGCTTTGG-3′). IL-10 (F: 5′-GCTCTTACTGACTGGCATGAG-3′; R: 5′-CGCAGCTCTAGGAGCATGTG-3′).

### Statistical analysis

Results in this study are expressed as mean ± SEM of independent experiments. Group comparisons were performed using one or two-way ANOVA and Tukey’s post hoc test. Differences were considered significant at *P* < 0.05. All statistical analyses were carried out using the Graph Pad Prism 6 program.

## Results

### Expression of WISP1 is enhanced in Liver Tissue after I/R

**Figure 1:**
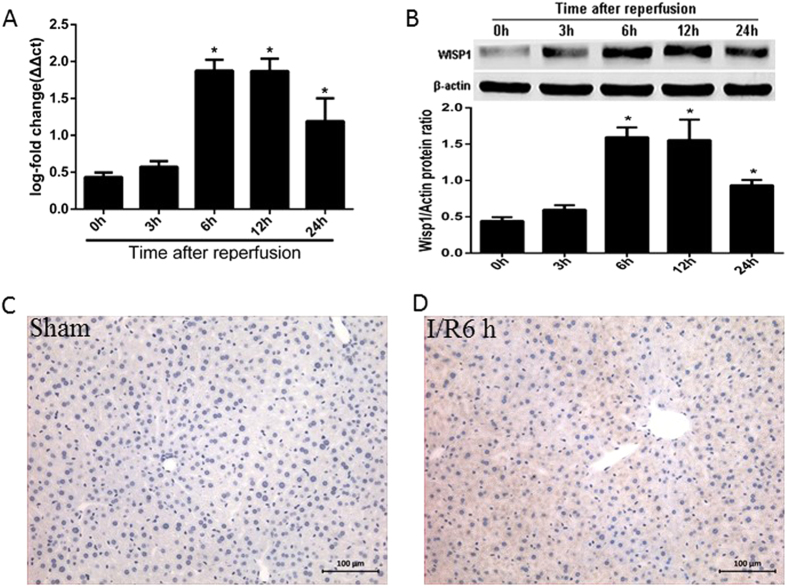
Expression of WISP1 is enhanced in liver tissue from mice after I/R. Mice underwent 60 min of ischemia and various times of reperfusion. Real-time quantitative PCR (RT-PCR) for liver WISP1 mRNA (**A**) and Western blot analysis for liver WISP1 protein (**B**) were performed for the ischemic lobes at the indicated time points, with each lane representing a separate animal. *P < 0.05 versus 0 h of reperfusion. (**C**) Immunohistochemistry stain of WISP1 from sections of sham group liver and (**D**) liver subjected to 60 min of ischemia and 6 h of reperfusion (×200 magnification). Significant difference from 0 h of reperfusion group. All the results are from at least three independent experiments; Data represent means ± SEM. Full-length blots are presented in Supplementary Figure S1.

To determine whether WISP1 participate in the liver tissue following I/R, real-time quantitative PCR and western blot were performed to detect WISP1 mRNA and protein levels. After 1h of warm ischemia, mice were sacrificed at 0 h, 3 h, 6 h, 12 h and 24 h of reperfusion, respectively (n = 6). WISP1 mRNA expression level was significantly up-regulated at 6 h and 12 h in I/R group (Fig. 1A, Supplementary Figure S1). Similar results were detected in WISP1 protein expression (Fig. 1B). Moreover, we detected WISP1 expression in liver tissue from mice that were subjected to hepatic I/R by immunohistochemistry. WISP1 was significantly expressed in mice liver following 6 h of reperfusion, when compared with sham group (Fig. 1C,D).

### Treatment with neutralizing monoclonal antibody to WISP1 decreases liver I/R injury

**Figure 2:**
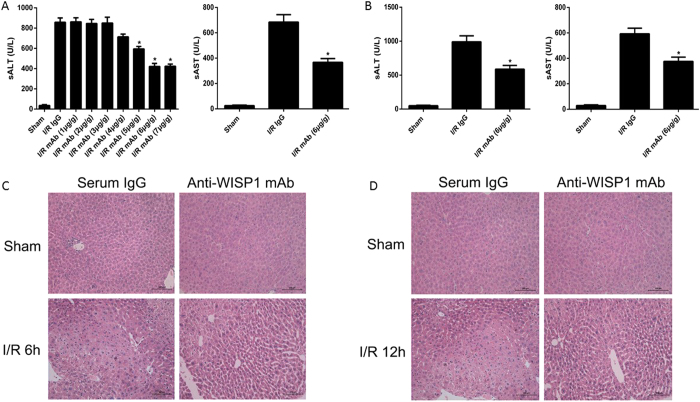
Treatment with neutralizing antibody to WISP1 decreased liver I/R injury. (**A**) Sham mice and mice that underwent ischemia and 6 h of reperfusion (I/R 6 h) were treated with anti-WISP1 antibody or control IgG antibody i.p. 1 h before ischemia and again at the time of reperfusion. Serum ALT levels were detected for hepatocellular injury. *P < 0.05 versus control IgG antibody treated group subjected to I/R. (**B**) Sham mice and mice that underwent ischemia and 12 h of reperfusion (I/R 12 h) were treated with anti-WISP1 antibody (6ug/g) or control antibody i.p. 1 h before ischemia and again at the time of reperfusion. *P < 0.05 versus IgG antibody treated group subjected to I/R. (**C**) Hematoxylin and eosin (H&E)–stained liver sections from control IgG antibody and anti-WISP1 antibody–treated mice 6 h and (**D**) 12 h after reperfusion (×200 magnification). All the results are from at least three independent experiments; Data represent means ± SEM.

To determine whether anti-WISP1 monoclonal antibody mediates liver I/R injury, mice were intraperitoneally administrated anti-WISP1 antibody or serum IgG antibody 1h before liver ischemia and again at the onset of reperfusion. One hour of liver ischemia followed by 6 h of reperfusion significantly enhanced serum alanine aminotransferase (ALT) levels in the IgG antibody treated mice that were suffered from I/R, when compared with sham group. Neither treatment with 1 μg/g nor 2 μg/g of anti-WISP1 antibody can alleviate hepatic I/R injury, whereas treatment with 6 μg/g of anti-WISP1 antibody showed a significantly protective effect in liver injury (Fig. 2A). This protective effect also emerged at 12 h after reperfusion in 6 μg/g anti-WISP1 antibody treated mice (Fig. 2B). Liver pathological changes were detected by hematoxylin-esoin-stained, and these results were concordant with the serum ALT or AST estimation of liver damage. Severe hepatocellular necrosis was emerged in liver tissue of mice that were treated with control serum IgG, whereas minimal damage was investigated in liver tissue from 6 μg/g anti-WISP1 antibody treated mice following 6 h of reperfusion (3.83 ± 0.41 vs 2.33 ± 0.52; n = 6, *p* < 0.05). Same results also emerged in the liver tissue from mice following 12 h of reperfusion (Fig. 2C,D).

### Treatment with anti-WISP1 monoclonal antibody decreases production of pro-inflammatory cytokines

**Figure 3:**
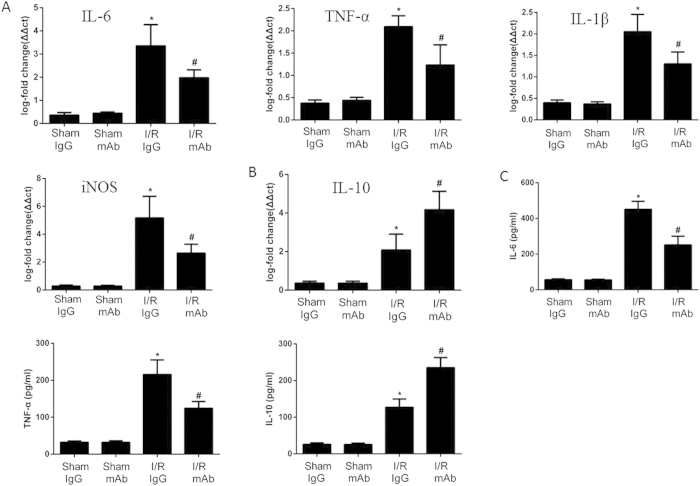
Treatment with anti-WISP1 antibody decreases production of liver inflammatory cytokines. The liver underwent 60 minutes of ischemia, followed by 6 h of reperfusion. (**A,B**) TNF-α, IL-6, IL-1β, iNOS and IL-10 mRNA expression in liver were detected by qPCR. (**C**) IL-6, TNF-α and IL-10 levels were assessed by ELISA. *P < 0.05 versus sham; ^#^P < 0.05 versus IgG treated group subjected to I/R. All the results are from at least three independent experiments; Data represent means ± SEM.

As is shown in Fig. 3, (A) IL-6, TNF-α, IL-1β, inducible nitric oxide synthase (iNOS) and (B) IL-10 mRNA levels were measured in the liver after I/R by real-time quantitative PCR. Compared with sham group mice, serum IgG treated group mice had a significantly higher IL-6, TNF-α, IL-1β and iNOS mRNA levels in liver following 6 h of reperfusion. However, mice that were treated with anti-WISP1 antibody showed significant decrease in liver IL-6, TNF-α, IL-1β and iNOS mRNA levels compared with isotype control IgG treated mice. In addition, circulating levels of IL-6, TNF-α and IL-10 were detected by ELISA (Fig. 3C). Taken together, anti-WISP1 antibody demonstrated to inhibit the expression of pro-inflammatory cytokines while promoting anti-inflammatory cytokines secretion.

### Treatment with recombinant WISP1 protein worsens liver I/R injury

**Figure 4:**
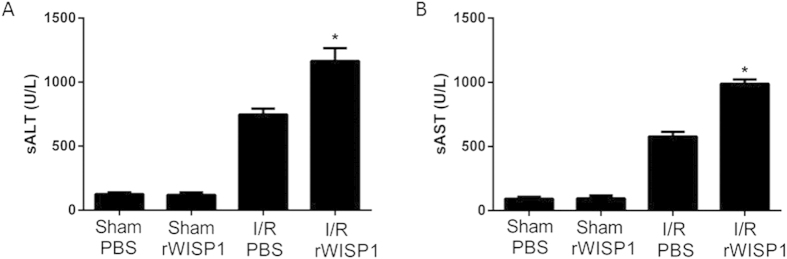
Treatment with recombinant WISP1 worsens liver I/R injury. Serum ALT (**A**) and AST (**B**) levels were measured in sham mice and mice that underwent 60 min ischemia and 6 h of reperfusion. Mice were treated with a nonlethal dose of recombinant WISP1 (20 ug) or sterile PBS immediately after reperfusion. *P < 0.05 versus PBS treated group subjected to I/R. All the results are from at least three independent experiments; Data represent means ± SEM.

In this section, we detect if exogenous WISP1 would affect liver injury in I/R, mice were made by injecting 20 μg (a nonlethal dose) recombinant WISP1 (rWISP1) i.p. at the beginning of reperfusion. There was no difference in liver injury as measured by sALT and sAST levels between sham animals that were given either control (PBS) or this secure dose of rWISP1 (Fig. 4A,B). As shown, liver I/R increased sALT and sAST levels in PBS-treated mice; however, mice that were given rWISP1 had a significantly greater increase in liver enzyme levels than controls.

### Treatment with recombinant WISP1 protein enhances production of pro-inflammatory cytokines

**Figure 5:**
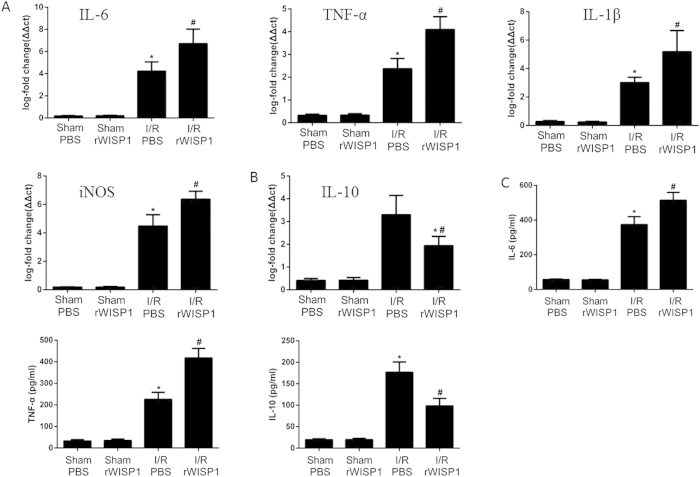
Treatment with recombinant WISP1 protein enhances production of inflammatory cytokines. The liver underwent 60 minutes of ischemia, followed by 6 h of reperfusion. (**A,B**) TNF-α, IL-6, IL-1β, iNOS and IL-10 mRNA expression in liver were detected by qPCR. (C) IL-6, TNF-α and IL-10 levels were assessed by ELISA. *P < 0.05 versus sham; ^#^P < 0.05 versus PBS treated group subjected to I/R. All the results are from at least three independent experiments; Data represent means ± SEM.

As is shown in Fig. 5, (A) IL-6, TNF-α, IL-1β, iNOS and (B) IL-10 mRNA levels were measured in the liver after I/R by real-time quantitative PCR. Compared with sham group mice, sterile PBS treated group mice had a significantly higher IL-6, TNF-α, IL-1β and iNOS mRNA levels in the liver following 6 h of reperfusion. However, mice that were treated with recombinant WISP1 protein showed significant increase in liver IL-6, TNF-α, IL-1β and iNOS mRNA levels compared with sterile PBS treated mice. Moreover, serum levels of IL-6, TNF-α and IL-10 were measured by ELISA (Fig. 5C). Together, recombinant WISP1 demonstrated to promote the expression of pro-inflammatory cytokines while constraining anti-inflammatory cytokines secretion.

### WISP1 mediated liver I/R injury depends on TLR4 signaling

**Figure 6:**
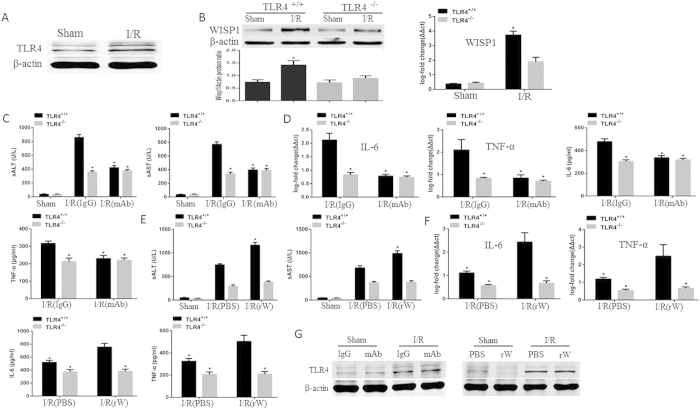
WISP1-mediated liver I/R injury depends on TLR4 signaling. The liver underwent 60 minutes of ischemia, followed by 6 h of reperfusion. (**A**) TLR4 protein levels were detected by western blot. (**B**) WISP1 levels were measured by western blot and qPCR in the liver of TLR4 intact (TLR4^+/+^) and TLR4 knockout (TLR4^−/−^) mice. *P < 0.05 versus TLR4^−/−^ mice subjected to I/R. (**C**) TLR4^+/+^ and TLR4^−/−^ mice were treated with anti-WISP1 antibody or negative control IgG. Serum ALT and AST were assessed. (**D**) IL-6 and TNF-α levels in the liver were detected by qPCR and the circulating levels were measured by ELISA. *P < 0.05 versus IgG treated group subjected to I/R. (**E**) TLR4^+/+^ and TLR4^−/−^ mice were treated with recombinant WISP1 protein (rW) or PBS. Serum ALT and AST levels were assessed. (**F**) IL-6 and TNF-α levels in the liver were detected by qPCR and the circulating levels were measured by ELISA. *P < 0.05 versus PBS treated group subjected to I/R. (**G**) Mice were treated with anti-WISP1 antibody, recombinant WISP1, or their negative control. TLR4 protein levels in the liver were measured by western blot. All the results are from at least three independent experiments; Data represent means ± SEM. Full-length blots are presented in Supplementary Figure S2 and S3.

Previous studies have shown TLR4 was required for I/R-induced injury in the liver and acted as a receptor for WISP1^15^,^29^. To determine if TLR4 was involved in WISP1-mediated I/R injury, western blot and PCR were used to detect TLR4 and WISP1 expressions. We found TLR4 expression was significantly increased in the liver tissue of mice subjected to I/R, when compared with sham group mice (Fig. 6A, Supplementary Figure S2A); WISP1 levels were significantly decreased in TLR4 knockout mice, when compared with wild-type mice following 6 h of reperfusion (Fig. 6B, Supplementary Figure S2B). To further investigate the role of TLR4 in WISP1 mediated liver I/R injury, TLR4 knockout (TLR4^−/−^) and TLR4 intact (TLR4^+/+^) mice were treated with anti-WISP1 antibody or recombinant WISP1 protein. Similar to the results mentioned above, blockade of WISP1 in TLR4^+/+^ mice led to protection from liver I/R damage by detecting serum ALT and AST levels (Fig. 6C). However, this protection was not found in TLR4^−/−^ mice subjected to liver I/R. We also detect the expression of IL-6 and TNF-α mRNA in the liver of these mice following I/R. Whereas TLR4^+/+^ mice that were treated with anti-WISP1 antibody showed decreased liver TNF-α and IL-6 mRNA levels compared with IgG group mice subjected to I/R, there were no significant difference in expression levels of these cytokines in TLR4^−/−^ mice following liver I/R (Fig. 6D).

Next, we administrated rWISP1 to TLR4^+/+^ and TLR4^−/−^ mice. Our results showed that there were no significant difference in serum ALT and AST levels in sham group mice that were injected with PBS or rWISP1 (Fig. 6E). Interestingly, rWISP1 worsened I/R injury in TLR4^+/+^ mice but had no effect in TLR4^−/−^ mice. And, similar results were seen by detecting inflammatory cytokines (Fig. 6F). Most importantly, neither in antiWISP1 nor in rWISP1 treated mice subjected to I/R could we found the significant change in the expression levels of TLR4, when compared with control groups (Fig. 6G, Supplementary Figure S3).

### WISP1 mediated liver I/R injury depends on TRIF signaling

**Figure 7:**
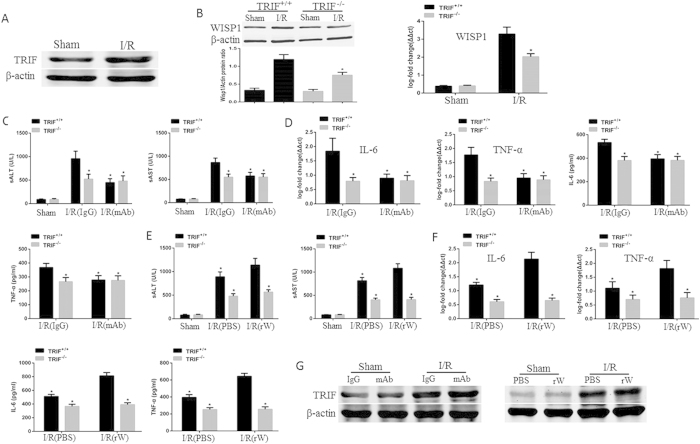
WISP1-mediated liver I/R injury depends on TRIF signaling. The liver underwent 60 minutes of ischemia, followed by 6 h of reperfusion. (**A**) TRIF protein levels were detected by western blot. (**B**) WISP1 levels were measured by western blot and qPCR in the liver of TRIF intact (TRIF^+/+^) and TLR4 knockout (TRIF^−/−^) mice. ^*^P < 0.05 versus TRIF^+/+^ mice subjected to I/R. (**C**) TRIF^+/+^ and TRIF^−/−^ mice were treated with anti-WISP1 antibody or IgG. Serum ALT and AST were assessed. (**D**) IL-6 and TNF-α levels in the liver were detected by qPCR and the circulating levels were measured by ELISA. *P < 0.05 versus IgG treated group subjected to I/R. (**E**) TRIF^+/+^ and TRIF^−/−^ mice were treated with rW or PBS. Serum ALT and AST levels were assessed. (**F**) IL-6 and TNF-α levels in the liver were detected by qPCR and the circulating levels were measured by ELISA. *P < 0.05 versus PBS treated group subjected to I/R. (**G**) Mice were treated with anti-WISP1 antibody, recombinant WISP1, or their negative control. TRIF protein levels in the liver were measured by western blot. All the results are from at least three independent experiments; Data represent means ± SEM. Full-length blots are presented in Supplementary Figure S4 and S5.

Previous study indicated that TLR4-mediated liver I/R injury depends on TRIF signaling^24^. In this part, we explored whether TRIF was involved in WISP1-mediated I/R injury, western blot and quantitative PCR were used to detect TRIF and WISP1 expressions. We found the expression of TRTF was significantly increased in the liver tissue of mice subjected to I/R, when compared with sham group mice (Fig. 7A, Supplementary Figure S4A); WISP1 expression was significantly decreased in TRIF knockout (TRIF^−/−^) mice, when compared with wild-type mice following 6 h of reperfusion (Fig. 7B, Supplementary Figure S4B). In addition, TRIF^−/−^ and TRIF intact (TRIF^+/+^) mice were treated with anti-WISP1 antibody or recombinant WISP1 protein. We found serum ALT and AST levels were significantly down-regulated in TRIF^−/−^ mice, compared with TRIF^+/+^ mice subjected to liver I/R. Importantly, Anti-WISP1 antibody or rWISP1 protein can significantly affect serum ALT and AST levels (Fig. 7C,E) and inflammatory cytokines (Fig. 7D,F) in TRIF^+/+^ mice following liver I/R, but not in TRIF^−/−^ mice. However, neither in antiWISP1 nor in rWISP1 treated mice subjected to I/R could we found the significant difference in the TRIF expression, when compared with control groups (Fig. 7G, Supplementary Figure S5).

### WISP1 regulates inflammatory cytokines secretion in kupffer cells

**Figure 8:**
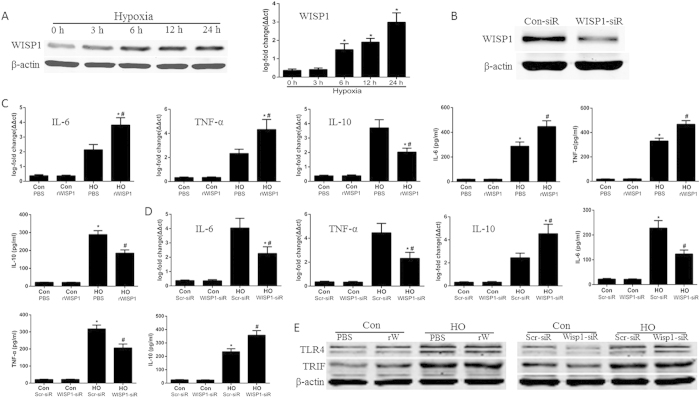
WISP1 regulates inflammatory cytokines secretion in kupffer cells. (**A**) KCs were treated with hypoxia at the indicated time points. WISP1 levels in the whole cell lysates were detected by western blot and qPCR. ^*^P < 0.05 versus 0 h exposure of hypoxia. KCs were treated with *WISP1*-siRNA (**B**) or negative control for 24 h, recombinant WISP1 protein (10 μg/ml) or PBS for one hour, and then exposure to hypoxia for 6 h. Inflammatory cytokines were measured by qPCR in whole cell lysates and by ELISA in cell supernatant (**C,D**). ^*^P < 0.05 versus control groups, ^#^P < 0.05 versus PBS or Con-siR treated groups subjected to hypoxia (HO). (**E**) Exposure of KCs to hypoxia for 6 h. TLR4 and TRIF levels in whole cell lysates were measured by western blot. All the results are from at least three independent experiments; Data represent means ± SEM. Full-length blots are presented in Supplementary Figure S6 and S7.

In this part, we intended to further explore how WISP1 could affect TLR4/TRIF signaling in mice primary kupffer cells (KCs) following hypoxia. We found exposure of the KCs to hypoxia (1% oxygen) caused a time-dependent increase in WISP1 expression in vitro (Fig. 8A, Supplementary Figure S6A). Next, we intended to disclosure the function of WISP1 in KCs response. Primary KCs were treated with rWISP1, *WISP1*-siRNA, or their control, respectively, following by hypoxia (Fig. 8B, Supplementary Figure S6B). The results showed that the expression of pro-inflammatory cytokines, including IL-6 and TNF-α, was significantly increased and the anti-inflammatory cytokine IL-10 was significantly decreased by rWISP1 treatment, either at gene or protein levels, in KCs following 6 hours of hypoxia (Fig. 8C). Conversely, knock down of WISP1 with the siRNA lead to downregulation of these pro-inflammatory cytokines and upregulation of IL-10 (Fig. 8D). However, we didn’t find any significant change in the expression of TLR4 and TRIF levels in KCs treated with rWISP1 or *WISP1*-siRNA following hypoxia (Fig. 8E, Supplementary Figure S7).

## Discussion

Hepatic ischemia reperfusion (I/R) injury is still a severe problem when patients undergo transplantation, elective liver resections or other damages, which may cause liver dysfunction, and even decrease the survival rate in the perioperative period^30^. Thus, ameliorating I/R induced injury is essential for patients’ postoperative recovery. Several harmful factors have been identified in I/R injury^6^,^7^,^8^,^9^. Recently, Li HH *et al.* have found a susceptibility gene to organ injury, WISP1, which was concluded as a damage associated molecule in ventilator induced acute lung injury^15^. Our further study have showed WISP1 might act as a harmful mediator in septic mice^16^. However, its role in hepatic I/R induced injury is still in the air. TLR4 signaling pathway play a pivotal role in various physiology and pathology processes, including warm liver I/R^31^. It can recognize some kinds of Danger-associated molecular patterns (DAMPs), such as HMGB1, fibronection and heat shock proteins. Although we have not yet determined whether WISP1 could be a DAMP molecule, in view of our recently findings^15^,^16^, we hypothesis that WISP1 might be also recognized by TLR4 signaling.

The purpose of this study was to uncover the notion that WISP1 contributes to liver I/R induced injury and depends on TLR4/TRIF signaling pathway. The major and novel findings of this study are: a) WISP1 levels are increased in the liver tissue from mice subjected to I/R and increased in the primary kupffer cells following hypoxia; b) blockade of WISP1 alleviates warm hepatic I/R induced injury; c) blockade of WISP1 decreases pro-inflammatory cytokines expression; d) recombinant WISP1 (rWISP1) worsens liver I/R injury and increases pro-inflammatory cytokines secretion; and e) WISP1-mediated hepatic I/R injury requires TLR4/TRIF signaling pathway.

WISP1 was originally uncovered as a Wnt1-responsive target, which contains the connective tissue growth factor, cysteine-rich-61 (CYR61) and nephroblastoma overexpressed (NOV)^32^,^33^. Previous studies about WISP1 mainly focused on chronic diseases, such as cancer and fibrosis. In this study, we sought to reveal the biological function of WISP1 in I/R induced acute liver injury. We showed that hepatic WISP1 expression was significantly increased at 6 h and 12 h after reperfusion of ischemia liver in this study. We also showed that blockade of WISP1 by using anti-WISP1 antibody can ameliorate hepatic I/R injury and decrease pro-inflammatory cytokine expression in ischemia liver tissue. However, it is unclear whether the increases in WISP1 levels are essential to the pro-inflammatory effects. WISP1 is a secreted extracellular protein, whereas it is not known whether WISP1 is secreted or passively released from damaged cells following I/R induced liver injury in our study. In addition, we have not found any significant increases about serum WISP1 levels in mice, which might reveal a local or paracrine-like action for WISP1.

To identify the molecular mechanisms and signaling transduction pathways, in which WISP1 is involved, we explored TLR4/TRIF signaling pathway. The Toll-like receptors (TLRs) play a pivotal role in the innate immune system against pathogenic microorganisms by recognizing the pathogen-associated molecular patterns^34^ or host-derived molecules by recognizing the danger-associated molecular patterns^35^, which have been discovered to involve in hepatocellular damage in liver I/R injury. TLR4 is one of the TLRs, which plays a vital role in endotoxin signal transduction and recognizes different kinds of endogenous ligands, such as fibronection, β-defensin, heat shock protein 72 and HMGB1^36^,^37^,^38^,^39^. The release of these ligands function as TLR4 agonists can stimulate inflammatory activity and modulate TLR4 signaling cascades. Our research support that the TLR4 receptor may also play an important role in WISP1-mediated liver I/R injury. WISP1 expression was significantly enhanced in TLR4^+/+^ mice that were subjected to hepatic I/R, while this enhancement was negated when detected in TLR4^−/−^ mice. Moreover, TLR4^−/−^ mice that were suffered from hepatic I/R are not affected by the administration of rWISP1 protein or anti-WISP1 antibody, when compared with the mice treated with isotype control IgG.

TRIF is a key adaptor, which located in the downstream of TLR4. TLR4 signaling through TRIF can result in the progress of various physiological and pathological changes^40^,^41^,^42^. TRIF also plays an important role in the pathogenesis of liver I/R injury. Zhai *et al*. have reported TLR4 mediated liver warm I/R damage mainly through TRIF signaling, but not MyD88 dependent^24^,^43^,^44^. We investigated the role of MyD88 in WISP1-mediated I/R injury and found depletion of MyD88 downregulated WISP1 expression in the liver, however, liver damage was not ameliorated in MyD88 knockout mice subjected to I/R (Supplementary Figure S8). Accordingly, our study mainly focused on TRIF signaling and also uncovered TRIF can significantly affect liver I/R injury. We also clarified WISP1 mediated liver I/R injury depends on TRIF signaling. However, neither attenuate nor heighten WISP1 could we found the decreased expression levels of TLR4 and TRIF in the liver following I/R. And this interesting phenomenon was also disclosed in vitro study by using primary kupffer cells suffered from hypoxia. Thus, we speculate that the secreted WISP1 from damaged liver tissue may produce the inflammatory injury response via activating the function of TLR4/TRIF, but not its expression.

In summary, our study suggests that WISP1 appears to be a damage associated molecule in hepatic I/R injury, and that the protective effects of blocking WISP1 and the detrimental effects of rWISP1 in hepatic I/R are dependent on the activation of the TLR4/TRIF function. Accordingly, blocking the abnormal expression of WISP1 may be a potential preventive therapy option against liver warm I/R injury.

## Additional Information

**How to cite this article**: Tong, Y. *et al.* WISP1 mediates hepatic warm ischemia reperfusion injury via TLR4 signaling in mice. *Sci. Rep.* **6**, 20141; doi: 10.1038/srep20141 (2016).

## Supplementary Material

Supplementary Information
