# Supplementary material for: WISP1 mediates hepatic warm ischemia reperfusion injury via TLR4 signaling in mice
Source: Sci Rep. 2016 Jan 29;6:20141. doi: 10.1038/srep20141 (PMC4731767; doi:10.1038/srep20141)
Supplement: Supplementary Information [file srep20141-s1.pdf]

WISP1 mediates hepatic warm ischemia reperfusion injury via TLR4 signaling in mice

Yao Tong[1*], Xi-Bing Ding[1*], Zhi-Xia Chen[1], Shu-Qing Jin[1], Xiang Zhao[1], Xin Wang[2], Shu-Ya Mei[3], Xi Jiang[1], Lingyu Wang[1], Quan Li[1]

[1]Department of Anesthesiology, Shanghai East Hospital, School of Medicine, Tongji University, Shanghai 200120, China;

[2] Department of Anesthesiology, First Clinical College of Nanjing Medical University, Nanjing 210029, Jiangsu, China;

[3]Department of Anesthesiology, School of Medicine, Nanchang University, Nanchang 330031, Jiangxi, China

*These authors contributed equally to this work.

Address correspondence to: Quan Li, Department of Anesthesiology, Shanghai East Hospital, School of Medicine, Tongji University, 150 Jimo Road, Shanghai 200120, China. Phone: +86- 021-65982875, Fax: +86-021-65982875, E-mail: quanligene@126.com

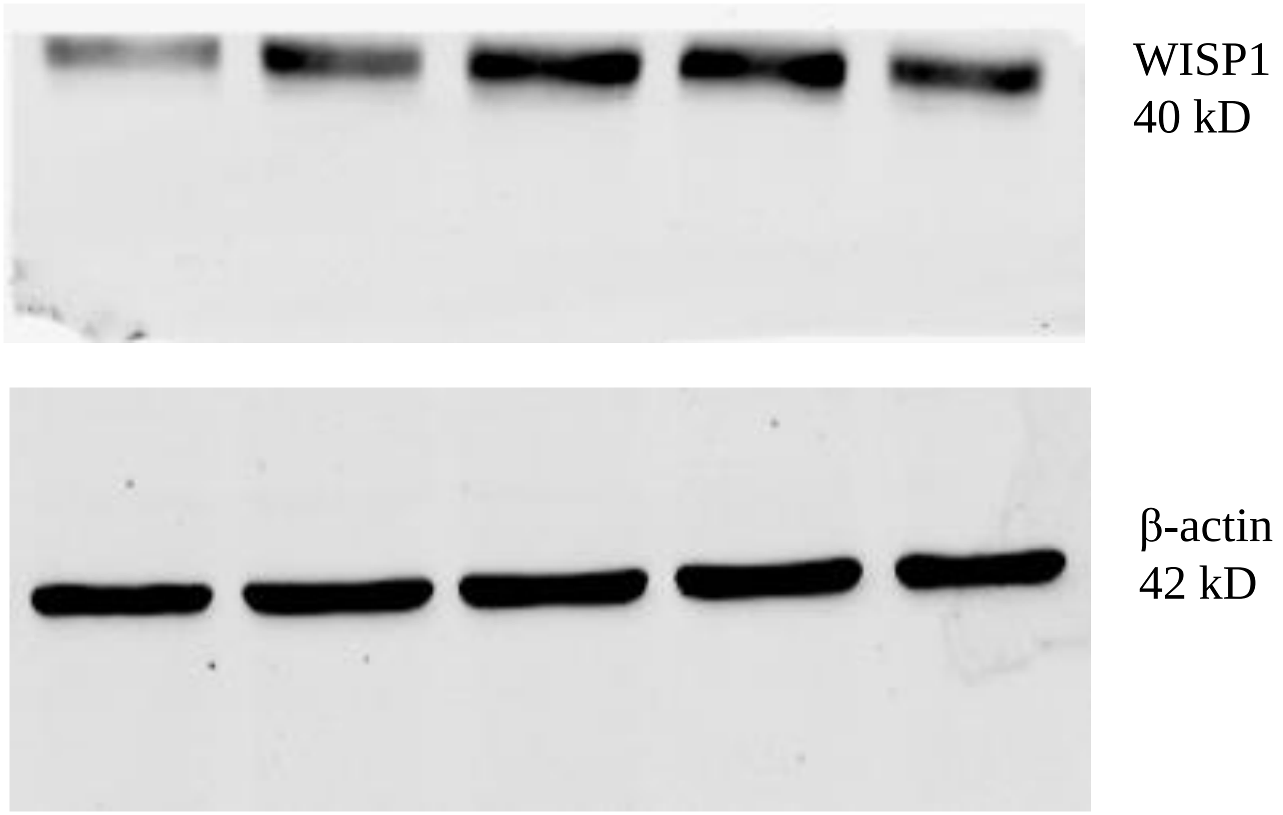

Supplementary Figure S1: Western blots showing the different WISP1 levels in the liver following indicated time points of reperfusion.

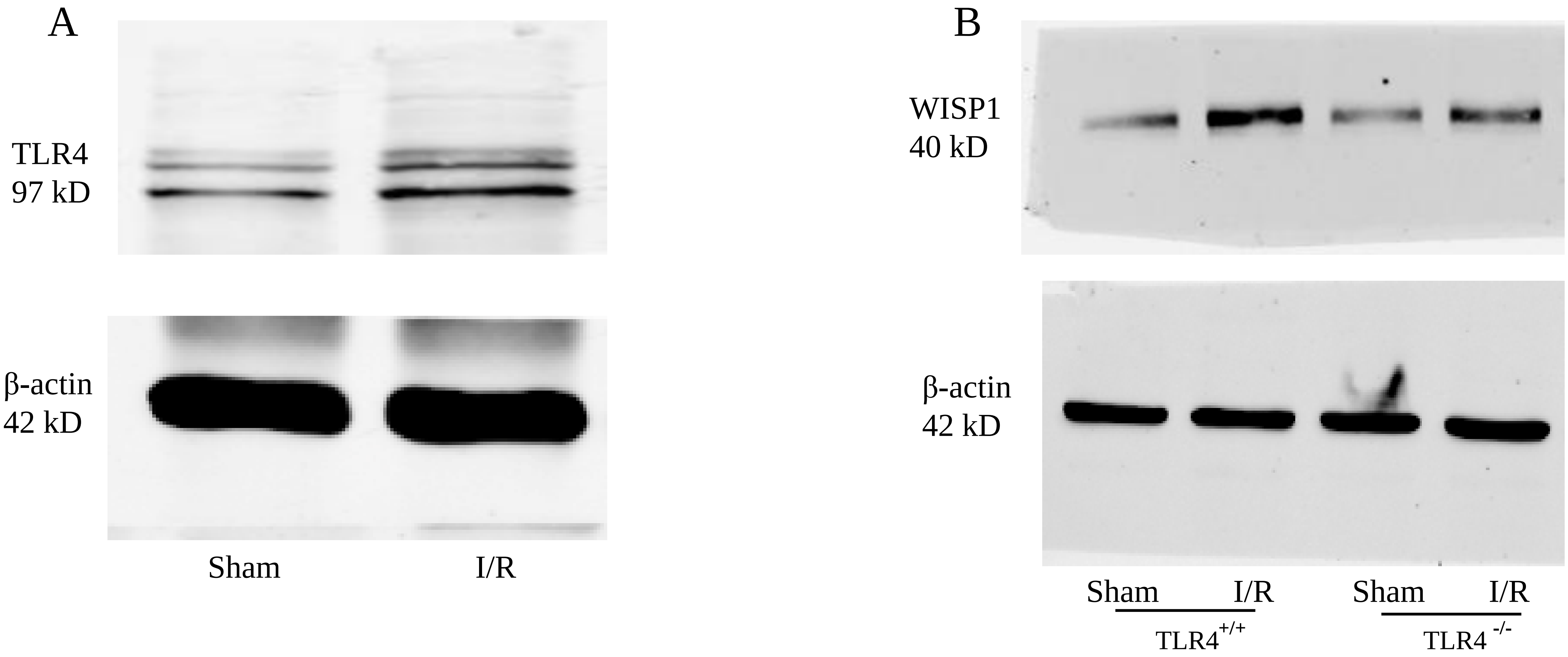

Supplementary Figure S2: (A)Western blots showing the different TLR4 levels in the liver of wild-type mice subjected to sham or I/R. (B) Western blots showing the different WISP1 levels in the liver of wild-type mice and TLR4 knockout mice subjected to sham or I/R.

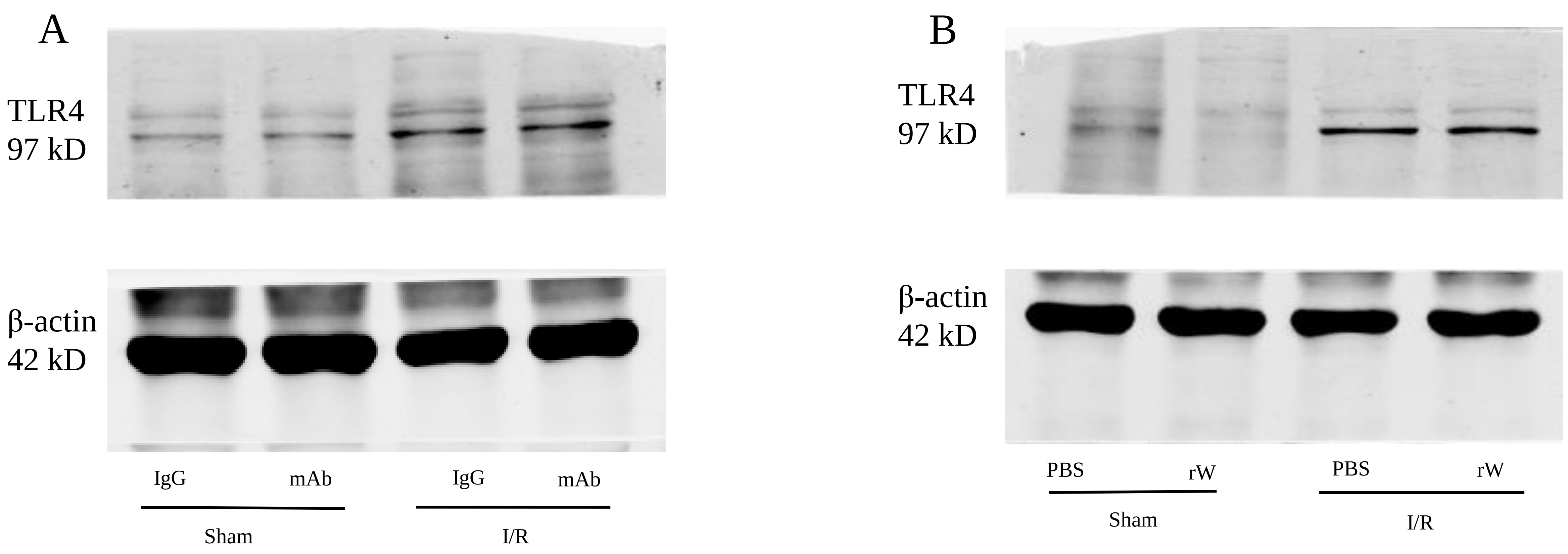

Supplementary Figure S3: (A)Western blots showing the different TLR4 levels in the IgG or mAb treated group subjected to I/R. (B)Western blots showing the different TLR4 levels in the PBS or rW treated group subjected to I/R. mAb: anti-WISP1 antibody; rW: recombinant WISP1 protein.

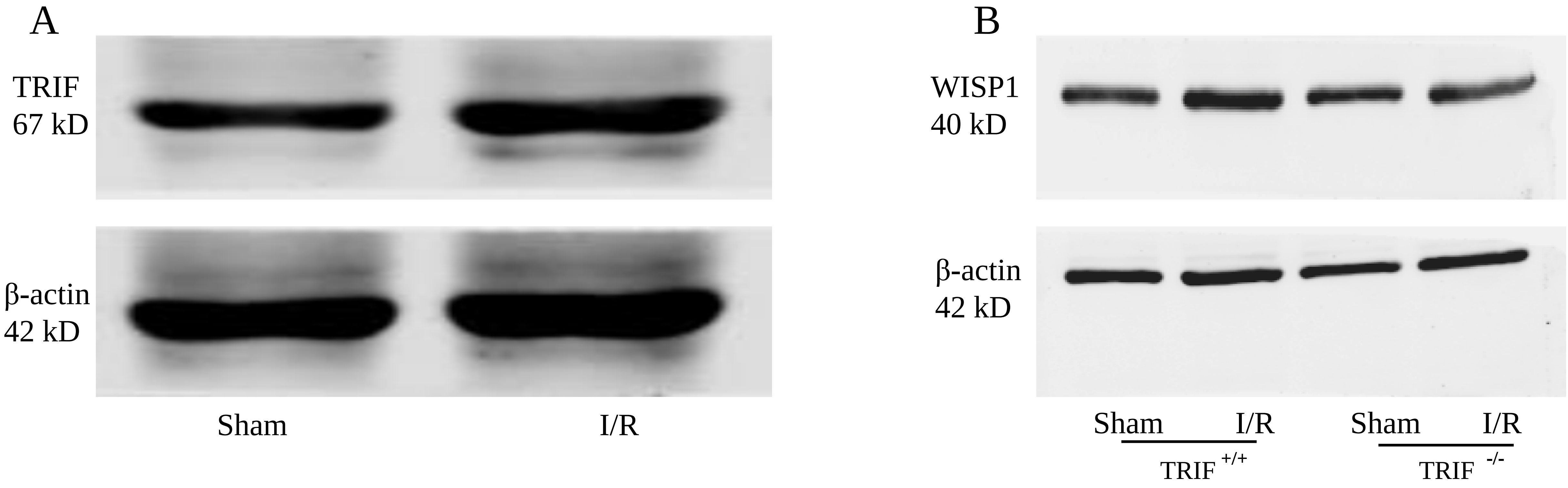

Supplementary Figure S4: (A)Western blots showing the different TRIF levels in the liver of wild-type mice subjected to sham or I/R. (B) Western blots showing the different WISP1 levels in the liver of wild-type mice and TRIF knockout mice subjected to sham or I/R.

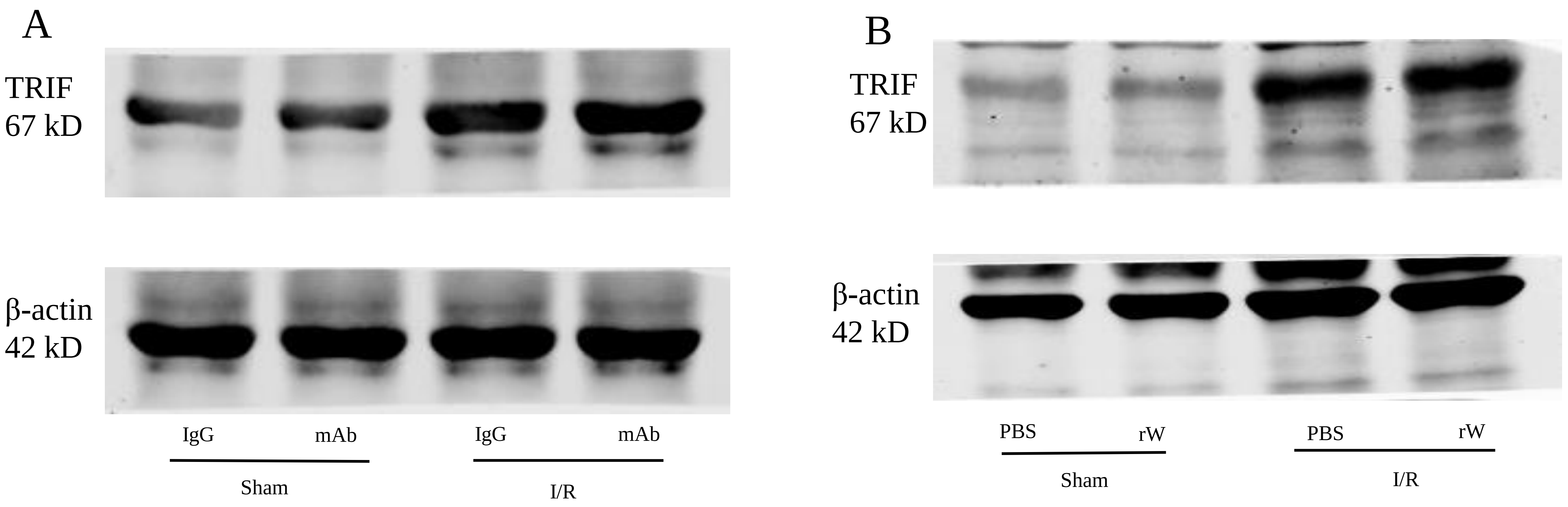

Supplementary Figure S5: (A)Western blots showing the different TRIF levels in the IgG or mAb treated group subjected to I/R. (B)Western blots showing the different TRIF levels in the PBS or rW treated group subjected to I/R. mAb: anti-WISP1 antibody; rW: recombinant WISP1 protein.

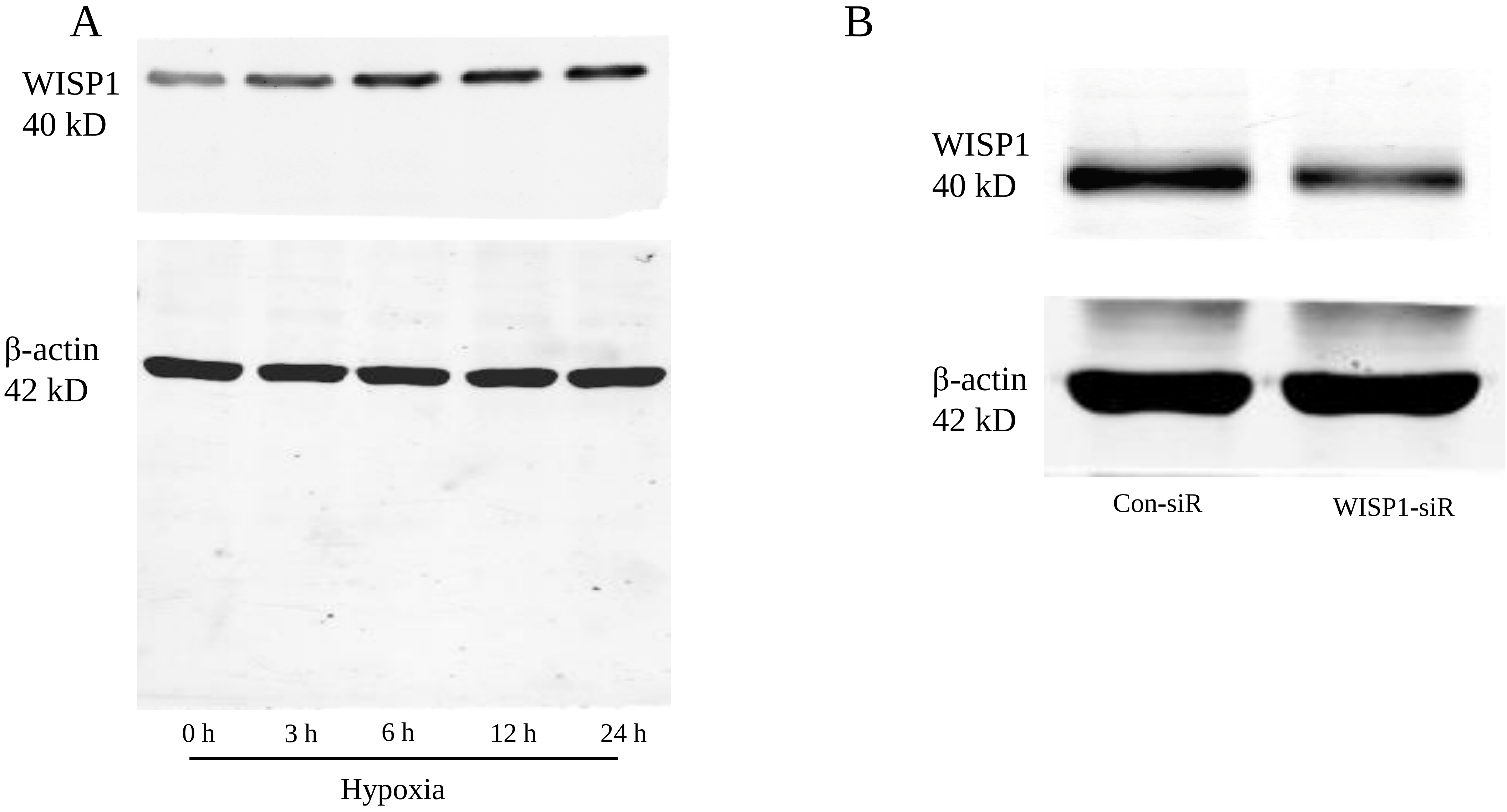

Supplementary Figure S6: (A) Western blots showing the different WISP1 levels in kupffer cells following indicated time points of hypoxia. (B) Western blots showing the different WISP1 levels in kupffer cells by siRNA.

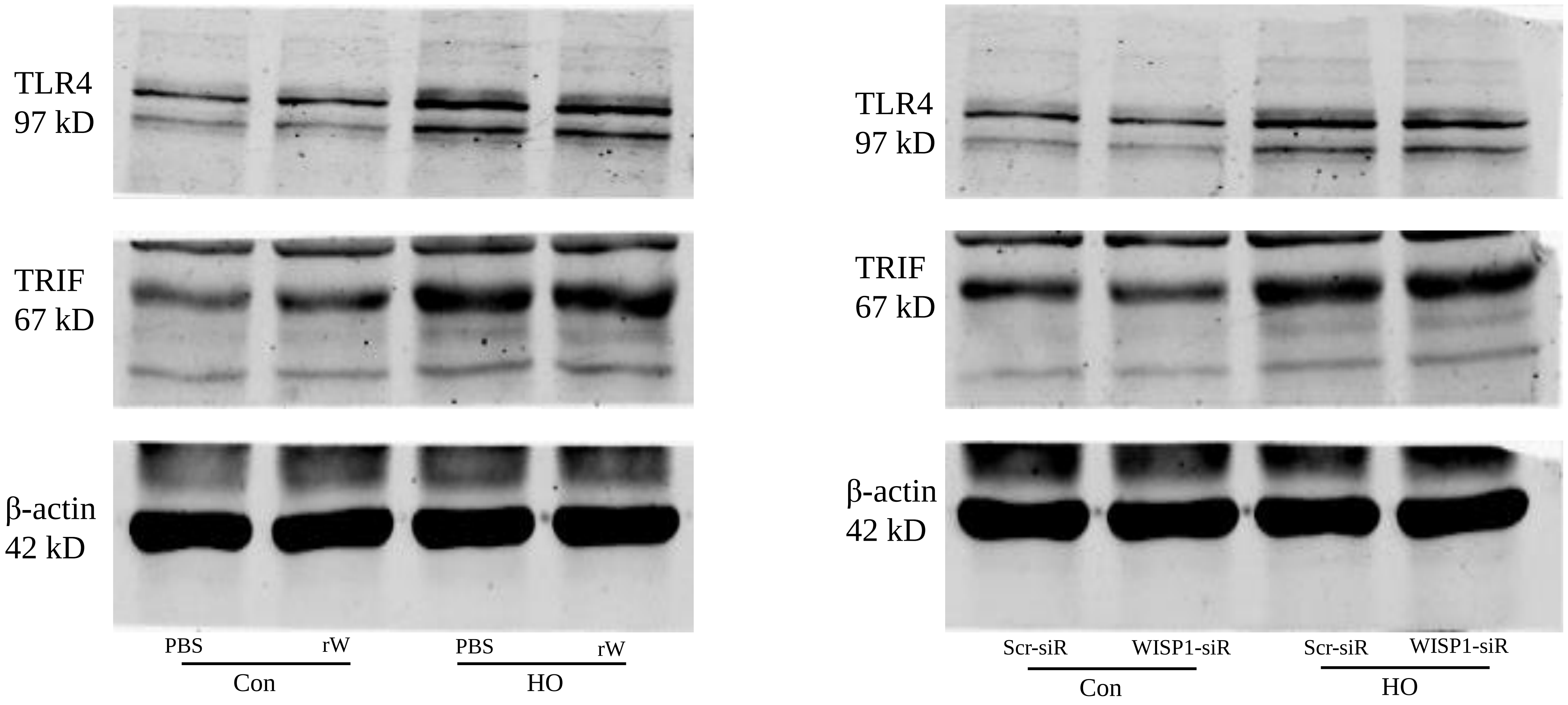

Supplementary Figure S7: (A) Western blots showing the different TLR4 and TRIF levels in the PBS or rW treated group following hypoxia. (B) Western blots showing the different TLR4 and TRIF levels in the kupffer cells following hypoxia by WISP1-siRNA. rW: recombinant WISP1 protein; HO: hypoxia.

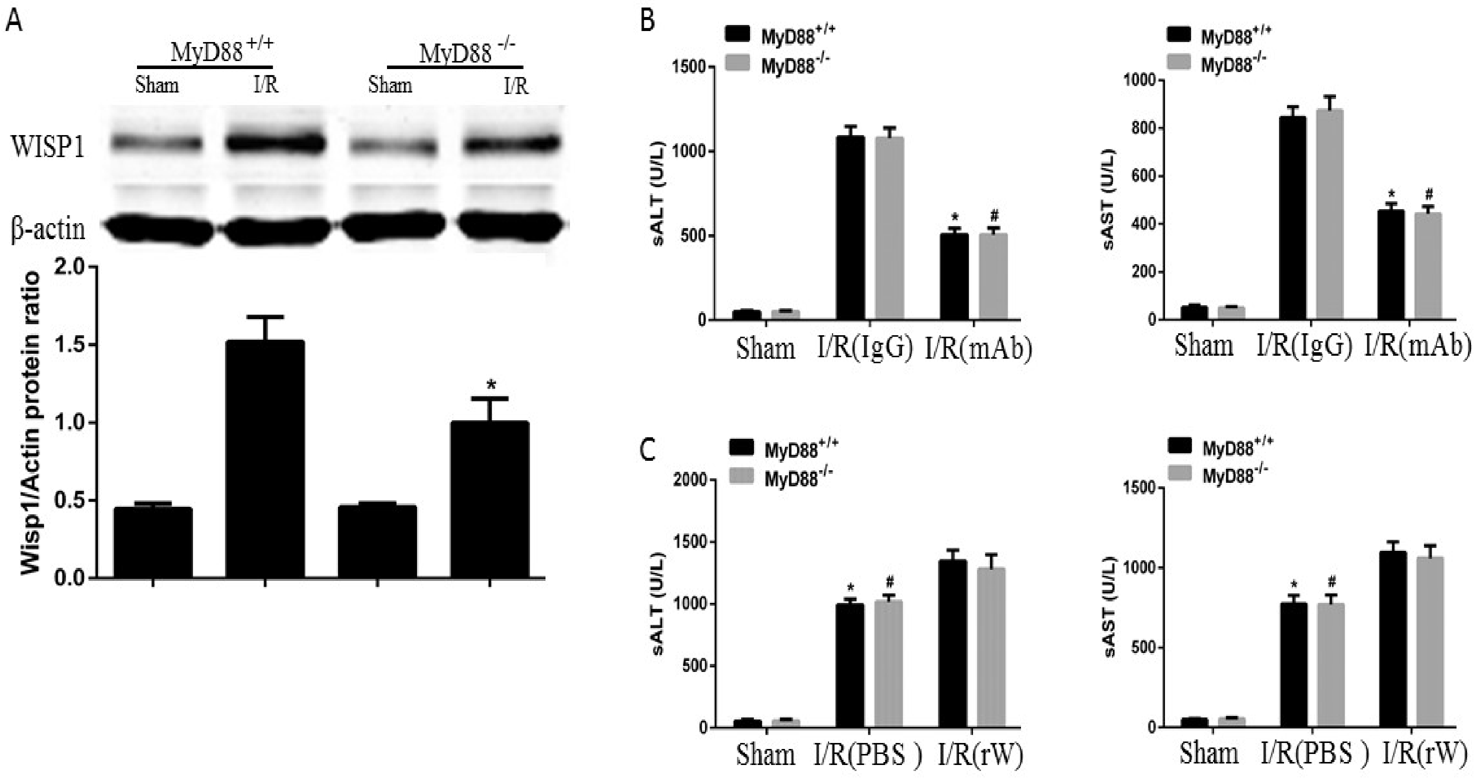

Supplementary Figure S8: WISP1 mediated liver I/R injury independent of MyD88 signaling. The liver underwent 60 minutes of ischemia, followed by 6 h of reperfusion. (A) WISP1 levels were measured by western blot in the liver of MyD88 intact ($MyD88^{+/+}$) and MyD88 knockout ($MyD88^{-/-}$) mice. *$P < 0.05$ versus $MyD88^{+/+}$ mice subjected to I/R. (B) $MyD88^{+/+}$ and $MyD88^{-/-}$ mice were treated with anti-WISP1 antibody (mAb) or negative control. Serum ALT and AST were assessed. *$P < 0.05$ versus IgG treated $MyD88^{+/+}$ mice subjected to I/R; $^{\#}P < 0.05$ versus IgG treated $MyD88^{-/-}$ mice subjected to I/R. (C) $MyD88^{+/+}$ and $MyD88^{-/-}$ mice were treated with recombinant WISP1 protein (rW) or negative control. Serum ALT and AST were assessed. *$P < 0.05$ versus rW treated $MyD88^{+/+}$ mice subjected to I/R; $^{\#}P < 0.05$ versus rW treated $MyD88^{-/-}$ mice subjected to I/R. All the results are from at least three independent experiments; Data represent means ±SEM.
